# Neuroprotective Effects of Isoquercetin: An *In Vitro* and *In Vivo* Study

**DOI:** 10.22074/cellj.2021.7116

**Published:** 2021-07-17

**Authors:** Qingxiao Yang, Zhichen Kang, Jingze Zhang, Fuling Qu, Bin Song

**Affiliations:** 1 Neurosurgery Department, Second Hospital of Jilin University, Changchun City, Jilin Province, 130000, China; 2 Rehabilitation Department, Second Hospital of Jilin University, Changchun City, Jilin Province, 130000, China

**Keywords:** Aβ Peptide, Alzheimer’s Disease, Antioxidant, Inflammation, Isoquercetin

## Abstract

**Objective:**

Alzheimer’s disease (AD) is considered a neurodegenerative disease that affects the cognitive function of elderly individuals. In this study, we aimed to analyze the neuroprotective potential of isoquercetin against the *in vitro* and *in vivo* models of AD and investigated the possible underlying mechanisms.

**Materials and Methods:**

The experimental study was performed on PC12 cells treated with lipopolysaccharide (LPS). Reactive oxygen species (ROS), antioxidant parameters, and pro-inflammatory cytokines were measured. In an *in vivo* approach, Wistar rats were used and divided into different groups. We carried out the Morris water test to determine the cognitive function. Biochemical parameters, antioxidant parameters, and pro-inflammatory parameters were examined.

**Results:**

The non-toxic effect on PC12 cells was shown by isoquercetin. Isoquercetin significantly reduced the production of nitrate and ROS, along with the altered levels of antioxidants. Isoquercetin significantly (P<0.001) down-regulated proinflammatory cytokines in PC12 cells treated with LPS. In the *in vivo* approach, isoquercetin- treated groups considerably showed the up-regulation in the latency and transfer latency time, as compared with AD groups. Isoquercetin significantly reduced Aβ-peptide, protein carbonyl, while enhanced the production of brain- derived neurotrophic factor (BDNF) and acetylcholinesterase (AChE). Isoquercetin significantly (P<0.001) reduced pro-inflammatory cytokines and inflammatory mediators, as compared with AD groups.

**Conclusion:**

Based on the results, we may infer that, through antioxidant and anti-inflammatory systems, isoquercetin prevented neurochemical and neurobehavioral modifications against the model of colchicine-induced AD rats.

## Introduction

Research suggests that age is the key factor for the development of acute and chronic neurodegenerative disorders, such as Alzheimer’s (AD), stroke, and Parkinson’s (PD) diseases (1). As we know that the aging process occurs on a global scale, the incidence of early mentioned diseases would be expectable in the near future (2, 3). Moreover, the available therapies only decelerate disease progression (2). Basic molecular science suggests that neuronal damage and death are both involved in the molecular mechanism of AD.* In vitro*, *in vivo*, and post-mortem investigations show neuronal death as a result of the activation of common cell death programs, such as apoptosis (3). Previous research suggests that the genetic manipulation or pharmacological interventions of underlying molecular pathways can protect neurons from deadly insults (4). The strategies for the treatment of neurodegenerative disorders are mainly focused on interrupting, antagonizing, or slowing the molecular events, leading to the irreversible injury or death of neurons in neurodegenerative diseases, which are commonly called neuroprotection (5). Nevertheless, poor translation of rodent investigations into clinical trials has overturned neurologists and provoked hot debates on the reasons for the apparent failure of neuroprotection against neurodegenerative diseases (4).

AD is considered a wide spread neurodegenerative disease, which affects the cognitive function of elderly individuals (6). In the course of AD, cortical neuron damage and disintegration of the hippocampal region cause memory impairment and alter the cognitive capability (4, 6). The 1^st^ clinical symptoms of AD is characterized by the demolition of the short-term memory. The pathological hallmark of AD is the presence of senile plaques, triggering the accumulation of β-amyloid proteins, through the deterioration of neurofibrillary tangles and neuronal and other proteins (6).

Amyloid β peptide (Aβ) has been considered a possible source of oxidative stress during AD (6, 7). It can generate free radicals that contribute to the expansion of toxic effects. Previous studies suggested that Aβ-induced cytotoxicity stimulates the accumulation of intracellular reactive oxygen species (ROS), finally leading to the peroxidation of membrane lipids and induced cell death. While the mechanism of Aβ-induced cytotoxicity is still unknown, previous investigations indicate that targeting Aβ would be regarded as a significant neuroprotective approach for the treatment and prevention of the onset of AD. Several lines of evidence suggest that antioxidant therapy has beneficial roles in the treatment of the toxic effects related to Aβ-induced oxidative stress (6, 8). Due to the lack of effective treatment for AD, researchers have focused their research on neuroprotective drugs, having antioxidant and anti-inflammatory properties.

Various efforts have been made in the world to explore the possible treatment for AD; however, there is no effective therapy for the cure of AD. Clinical research suggests that most of the AD patients over the age of 65 suffer from the disease; nevertheless, the symptoms can occur at the early stages (6). The aetiology of AD is very complex, and various pathways of neuronal injury have been proposed (9). According to previous studies, the cortex, hippocampus, and limbic system are considered susceptibility regions for the injury (6, 10). Studies showed that Aβ peptide might be a possible source for the induction of oxidative stress in the brain of AD patients (2, 6). It is now known that it can generate free radical agents that participate in the expansion of its side/toxic effects. Research suggests that Aβ-induced cytotoxicity stimulates the intracellular accumulation of neuronal plaques as a result of the generation of oxidative stress, ultimately leading to the peroxidation of lipids and induction of cell death (2, 6). However, the precise mechanism underlying the role of Aβ-induced neurotoxicity is still unclear. The prevention of Aβ accumulation with in the brain is one of the significant targets for the potential therapy of AD, as numerous drugs are screened for this ability as to whether they can halt the onset of the disease (4, 9). Consequently, the antioxidant therapy is the best approach for reducing the pathological conditions and side/toxic effects linked with Aβ-induced oxidative stress. Due to the beneficial effects of antioxidant compounds, researchers have devoted themselves to discovering novel phytochemical agents having antioxidant potential to modulate the detrimental effects of Aβ-induced neurotoxicity.

Isoquercetin (quercetin-3-O-b-D-glucopyranoside) is found in various medicinal and culinary crops, including fruits, herbs, and vegetables (11). The anti-allergic, antioxidant, and anti-inflammatory activities of isoquercetin against different rodent models a real ready documented (12, 13). Paulke et al. (14) showed higher bioavailability of isoquercetin in comparison with quercetin. To the best of our knowledge, the impact of isoquercetin on the prevention of cognitive defects occurring in AD patients has not been studied. In the current experimental study, we aimed to assess the neuroprotective potential of isoquercetin against the *in vitro* and *in vivo* models of AD to explore the possible mechanism.

## Materials and Methods

### Chemicals

Dulbecco’s modified Eagle’s medium (DMEM) was procured from the Thermo Fisher Scientific, Inc, Waltham, MA, USA.

### *In vitro* study

### Cell culture

In this experimental study, the PC12 (phaeochromo-cytoma) cell line was purchased from the Shanghai Biochemistry Co., Ltd (Shanghai, China). High-glucose DMEM was used for the culture of the cells containing streptomycin (10 U/ml), fetal bovine serum (10%), and penicillin (100 U/ml). The cells were incubated in an incubator at 37o C in a humidified 95% air, 5% CO atmosphere. When the cells reached 80% confluency, they were treated with the trypsin (0.25%) and passaged.

### Cytotoxicity assay

The method used for the determination of MTT assay was based on a method, as previously described with minor modifications (15). Briefly, the cells were seeded on to the 96-well plate at a density of 1×10^4^ cells/plate and further incubated at 37˚C for 24 hours in a CO_2_ chamber. After 24 hours, the medium was changed with a new medium, containing different doses of isoquercetin and incubated for 24 hours. After the incubation period, the medium (containing isoquercetin) was replaced with the MTT (200 μl) solution and incubated at 37˚C for the next 4 hours. After that the MTT solution was changed with dimethyl sulfoxide (DMSO) to solubilize the formazan crystals and incubation for 20 minutes at 37˚C with occasionally shaking, the absorbance of samples was read at 570 nm using a microplate reader (Thermo Scientific Multiscan GO, USA) and the cell viability was calculated (%). 

### Nitrite assay

The Griess assay was used for the determination of nitrite in accordance with a previously described method with minor modifications (16). Briefly, the cell supernatant (100 μL) was mixed with the Griess reagent (100 μL), containing nephtylethylenediamine (0.01%), p-amino-benzene sulphonamide (1%) in phosphoric acid (2.5% v/v) and kept the reaction mixture in the darkroom for 20 minutes. Finally, the optical absorbance was recorded at 570 nm using a microplate reader. 

### Estimation of reactive oxygen species generation

The Nitro Blue Tetrazolium (NBT) assay was applied for the measurement of ROS (16). Briefly, the cells were incubated with NBT and various concentrations of isoquercetin for 2 hours in the 96-well plates. The formazan crystals were solubilised using 2 M KOH (freshly prepared) in DMSO, and finally, the absorbance of specimens was monitored at 630 nm using a microplate reader. 

### The concentration of malondialdehyde

The concentration of malondialdehyde (MDA) was assayed, as previously described, with minor modifications (17). Briefly, the cells were seeded in a 6-well plate at a density of 4×10^5^ cells/well and treated with different concentrations of isoquercetin for 24 hours. Next, the cells were washed with the ice-cold phosphate buffer saline phosphate buffer saline (PBS) and scrapped in a sodium phosphate solution containing Triton-X (0.1%). The cells were lysed and finally centrifuged at 10,000 g for 10 minutes. Afterward, the resulting pellet was discarded, and the supernatant was collected. The cell supernatant (100 μL) was mixed with trichloroacetic acid (TCA, 10% w/v) and thiobarbituric acid (TBA, 0.67% w/v) and kept at 95˚C for 1hour. Then, the solution was quickly cooled and mixed with n-butanol-pyridine (15:1) and centrifuged for 10 minutes at 400 g. The upper layer (pink) was collected, and the absorbance was read at 532 nm using a microplate reader. 

### Determination of glutathione

Indirect estimation of oxidative injury, reduced glutathione (GSH) level was determined (17) Briefly, PC12 cells at a density (4×10^5^ cells/well) were propagated into 6-well plates and incubated at 37˚C for 24 hours in the CO_2_ chamber. Afterward, the cells were treated with the different concentrations of isoquercetin and washed with the ice-cold PBS and scrapped in sodium phosphate buffer containing Triton-X (0.1%). For the removal of proteins, the cells were lysed (100 μL) via adding trichloroacetic acid (10%) and again incubated at 4˚C for 1 hours and finally centrifuged at 5000g rpm for 5 minutes. After that, PBS (100 μl) and DTNB (50 μl) were mixed in the above supernatant (75 μl) and finally estimated the absorbance at 412 nm after the 10 minutes. 

### Catalase estimation

The method employed for the measurement of the catalase (CAT) enzyme activity was previously described, with minor modifications (17, 18). Briefly, the cell lysate was mixed into the hydrogen peroxidase (H_2_ O_2_) phosphate buffer solution in an Eppendorf tube, which was further vortexed and incubated at 37˚C for 3-5 minutes. After that, the resulting solution was mixed with the dichromic acetate solution and kept for 10 minutes at 100˚C. After that, the reaction mixture was cooled down using the tap water and centrifuge at 2500 g rpm for 5 minutes and the absorbance of specimens was read at 570 nm. 

### Superoxide dismutase activity

The method employed for the measurement of the superoxide dismutase (SOD) enzyme activity was previously described, with minor modifications (16- 18). Briefly, cell lysates (100 μM) was mixed with Tris buffer (1 ml). After that, pyrogallol was mixed, and the absorbance of the samples was read at 420 nm using a microplate reader for 5 minutes at a time interval of 1 minute. Finally, the activity of the SOD enzyme was reported as the percentage of inhibition of pyrogallol auto-oxidation.

### *In vivo* study

### Experimental animals

Swiss Wistar rats (150-180 g, either both sex) were used for the current protocol. The rats were kept in the polyethylene cages and kept standard laboratory conditions, such as temperature (22 ± 2o C), relative humidity (45-75%), and light/dark cycle (12/12 hours). The rats received the standard food pellets and water ad libitum. All the protocol was approved by The Institutional Ethical Committee (SHJU/19/01/05). 

### Experimental protocols

For the AD, the rats were grouped into the following groups, and each group had 12 animals as follows;

Normal control group rats (received vehicle only): group I

AD control received colchicine (15 µg/5 µl icv): group II

AD control received colchicine (15 µg/5 µl icv) and Isoquercetin (10 mg/kg): group III

AD control received colchicine (15 µg/5 µl icv) and Isoquercetin (20 mg/kg): group IV

AD control received colchicine (15 µg/5 µl icv) and Isoquercetin (40 mg/kg): group V

AD control received colchicine (15 µg/5 µl icv) and Memantine HCL (10 mg/kg): group VI

The rats were intracerebroventricularly infused with either artificial cerebrospinal fluid. Post-operative procedures or colchicine (15 Ig) mixed in the ACSF. After that, the animals were further used for the determination of neurobehavioral and neuro-chemical parameters (19). 

### Post-operative procedures

After the surgery, the rats were kept in the aseptic condition and received the standard diet (water and food), and the animals were treated with 5 mg/kg gentamicin (intraperitoneal injection) for the next three days to prevent the sepsis.

### Morris maze test

Morris maze test was used for the estimation of behavioural effect using the previous method with minor modifications (19). Briefly, a circular pool (180 cm in diameter and 60 cm in height) was used for the Morris maze test. The circular pool filled with the water and the entire experimental animal put into quadrants during the acquisition and retention phase (19, 20).

### Probe trial

For the probe trial, on the last day of training, the platform was removed from the pool, and experimental rats were left free to swim in the pool for the next 2 minutes (19). The time interval of the experimental animal reached the target quadrant was compared to other groups, and the data were shown as the latency time ± standard error means (SEM).

### Passive avoidance paradigm

The main purpose of the passive avoidance paradigm is determination the learning and memory capacity of the experimental animals (19). Briefly, the experimental rats were kept in the shuttle box, having 2 compartments (one for the light, while another for the dark) and unglued with a guillotine door. During the experiment, the rodent kept for 30 seconds in the light chamber and the next open a guillotine door and finally the experimental rodent were transferred into the dark chamber, and finally, closed the door for the next 10 seconds. 

### Neurochemical parameters

At the end of the experimental study, the neurochemical parameters, including protein carbonyl, acetylcholine esterase were estimated using the previously published literature with minor modifications (19).

### Antioxidant parameters

Antioxidant enzymes viz., CAT, GSH, lipid peroxidation (LPO) and SOD were estimated in the hippocampus via using the previously reported literature (19, 21).

### Inflammatory mediators

Pro-inflammatory cytokines like interleukin-1β (IL-1β), IL-6 and tumour necrosis factor-α (TNF-α) and inflammatory mediator including nuclear transcription factor (NF-κB), was estimated using standard kits.

### Statistical analysis

One-way analysis of variance (ANOVA) was used for the determination of statistical significance. The difference between the examined and control cells was analysed using the post hoc Newman-Kuels test, and the values are presented as mean ± SEM. P<0.05 was considered as a statistically significant value.

## Results

### Effect of isoquercetin on cell viability

**Fig.1:**
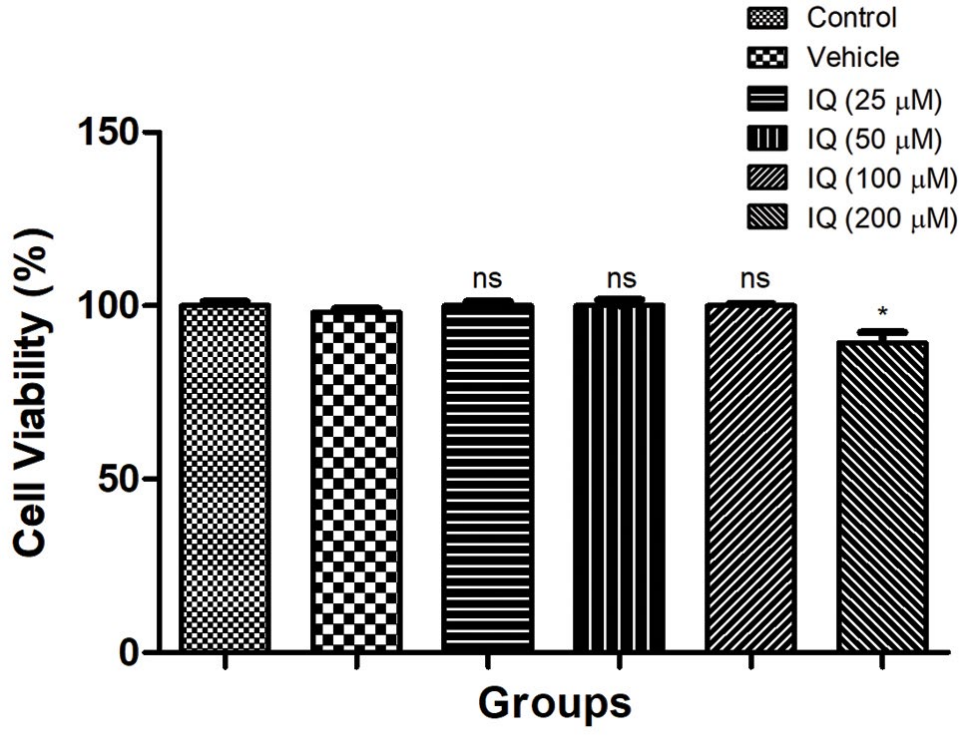
The cell viability effect of isoquercetin on P12 cells. ns; Non significant, IQ; Isoquercetin, and *; P<0.05.

Figure 1 exhibited the effect of isoquercetin on cell viability. Figure 1 exhibited that the isoquercetin 100 μM was found to be non-toxic as they did not induce any considerable alteration in the growth of PC12 cells. Moreover, isoquercetin dose up to 100 μM exhibited a sign of cytotoxicity with considerable change. Accordingly, isoquercetin at a dose range of 25-100 μM is a safe drug and can be used for further experimental analyses.

### Effect of isoquercetin on lipopolysaccharide induced nitrosative stress

**Fig.2:**
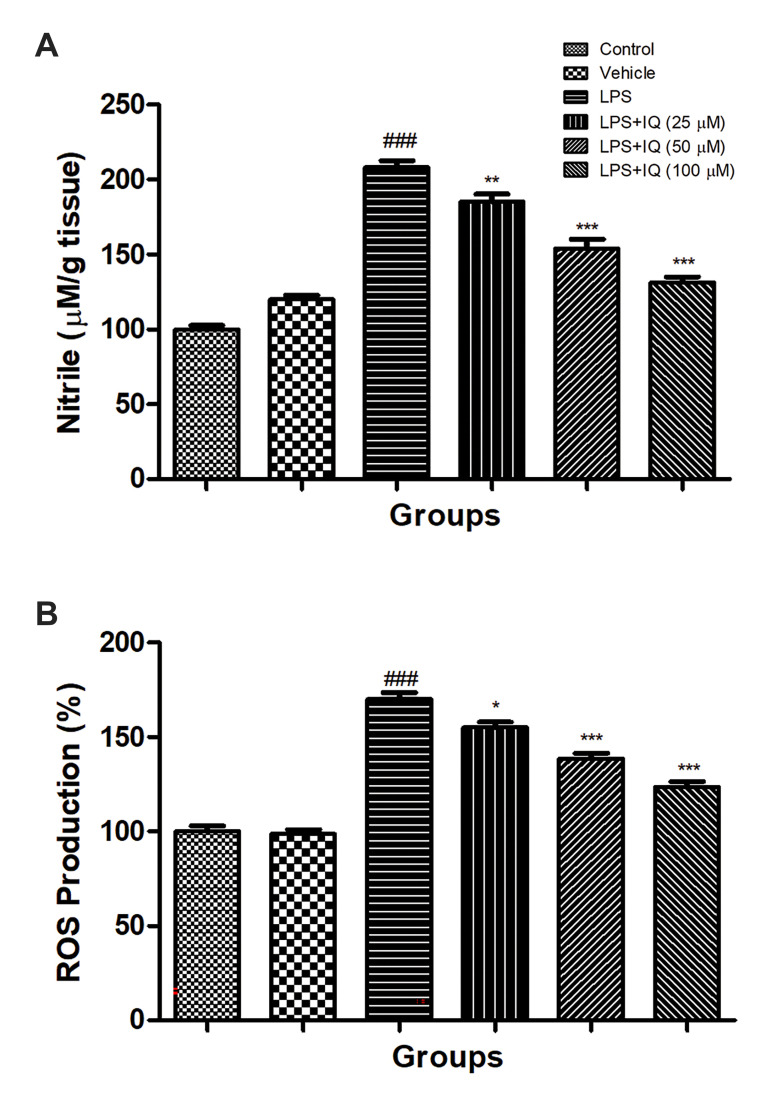
Effect of isoquercetin on the LPS induced nitrite and ROS activity in PC12 cells. **A. **Nitrite and **B. **ROS as described in the material and methods. Values are presented as mean ± SEM. ^### ^showed the significance as compared to the control group (P<0.001). * demonstrates the significance as compared to LPS-induced group (*; P<0.05, **; P<0.01, and ***; P<0.001). LPS; Lipopolysaccharide, IQ; Isoquercetin, and ROS; Reactive oxygen species.

It is well known that during the oxidative stress increased the nitrosative stress due to the generation of free radicals. A similar result was found in the current experimental study. PC12 cells were treated with lipopolysaccharide (LPS, 100 ng/mL) and showed the increased level of nitrite released into the supernatant (24 hours) as compared to the control. Isoquercetin treatment significantly (P<0.05) reduced the release of nitrite into the supernatant in a dose-dependent manner as compared to the LPS-stimulated cells (Fig.2A). 

### Effect of isoquercetin on lipopolysaccharide induced reactive oxygen species level

During AD, the ROS level considerably increased due to the generation of free radicals. PC12 cells treated with the LPS (100 ng/mL) considerably (P<0.001) boosted the production of ROS as compared to the normal cells. The concentration-dependent treatment of isoquercetin significantly (P<0.001) down-regulated the production of ROS as compared to the LPS treated cells (Fig.2B).

### Effect of isoquercetin on lipopolysaccharide induced antioxidant enzymes

**Fig.3:**
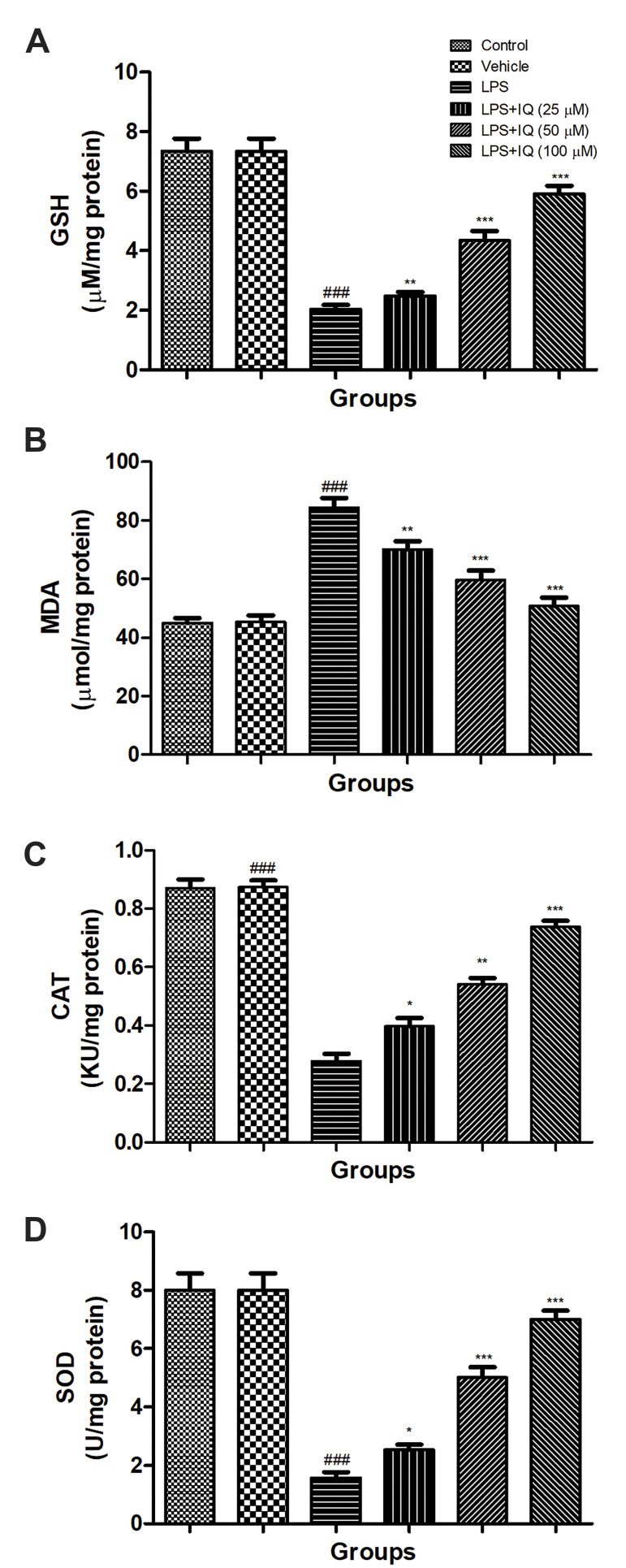
Effect of isoquercetin on the LPS induced antioxidant activity in PC12 cells. **A. **GSH, **B. **MDA, **C. **CAT and **D. **SOD as described in the material and methods. Values are presented as mean ± SEM.^ ###^ showed the significance as compared to the control group (P<0.001). * demonstrates the significance as compared to LPS-induced group (*; P<0.05, **; P<0.01, and ***; P<0.001). LPS; Lipopolysaccharide, GSH; Glutathione, MDA; Malondialdehyde, CAT; Catalase, SOD; Superoxide dismutase, and IQ; Isoquercetin.

LPS (100 ng/mL) treatment demonstrated the increased level of MDA as compared to healthy control cells. Isoquercetin treatment significantly (P<0.001) decreased the level of MDA as a dose-dependent manner as compared to LPS control cell lines (Fig.3A).

In the level of SOD, CAT and GSH, LPS (100 ng/ mL) treatment significantly (P<0.001) showed the reduced level as compared to normal cell lines, and the concentration-dependent treatment of isoquercetin exhibited the increased level of SOD (Fig.3B), CAT (Fig.3C) and GSH (Fig.3D) as compared to the LPS control cell lines. 

### Effect of isoquercetin on lipopolysaccharide induced pro-inflammatory cytokines

**Fig.4:**
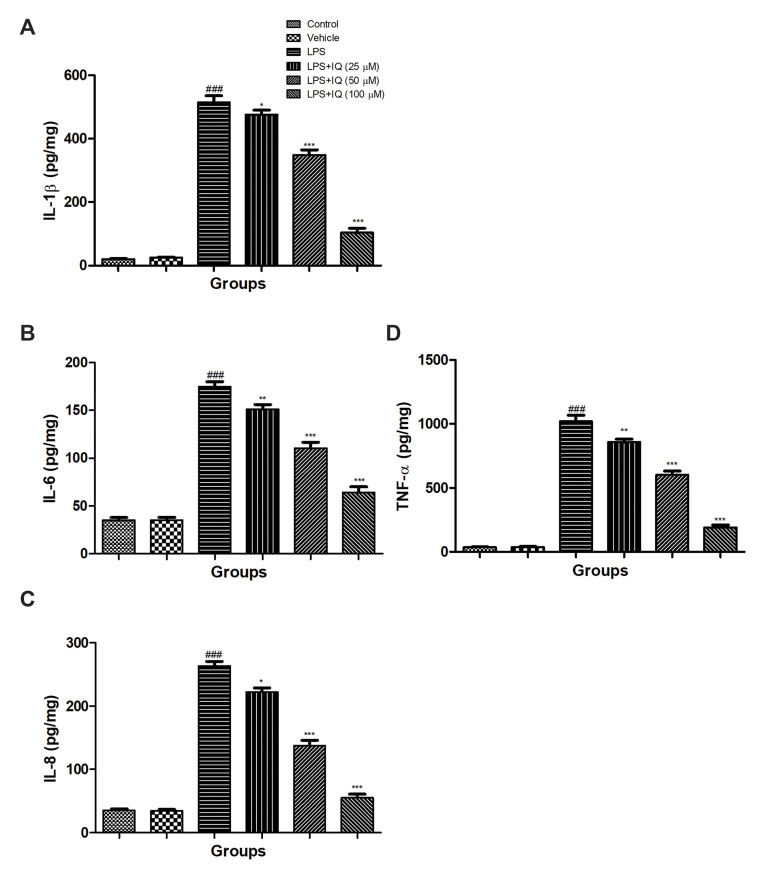
Effect of isoquercetin on the LPS induced pro-inflammatory cytokines parameter in PC12 cells. **A.** IL-1β,** B.** IL-6, **C.** IL-8 and** D.** TNF-α as described in the material and methods. Values are presented as mean ± SEM.^ ### ^showed the significance as compared to the control group (P<0.001). * demonstrates the significance as compared to LPS-induced group (*; P<0.05, **; P<0.01, and ***; P<0.001). LPS; Lipopolysccahride, IQ; Isoquercetin, IL-8; Interleukin-8, and TNF-α; Tumor necrosis factor-α.

Figure 4 showed the effect of isoquercetin and LPS on the pro-inflammatory cytokines on the PC12 cells. LPS (100 ng/mL) showed the significantly (P<0.001) increased the level of pro-inflammatory cytokines such as IL-1β, IL-6, IL-8, and TNF-α as compared to the control cell lines. The dose-dependent treatment of isoquercetin significantly (P<0.05) reduced the level of pro-inflammatory cytokines, including IL-1β (Fig.4A), IL-6 (Fig.4B), IL-8 (Fig.4C) and TNF-α (Fig.4D) as compared to the LPS induced control cell lines. 

### Time spent in the platform quadrant

**Fig.5:**
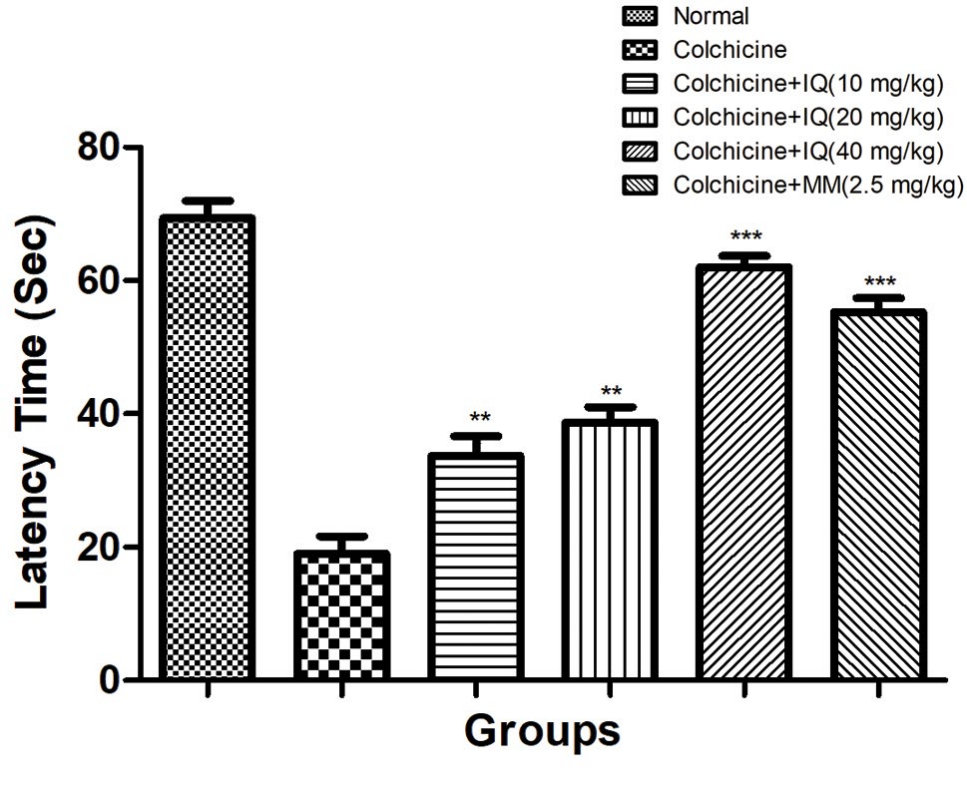
Effect of isoquercetin on the colchicine impaired spatial memory and learning in rats appraises via probe trial in water maze; data shows mean ± SEM of 6 rats in each group. Significant difference represent as compared to colchicine control group rats (**; P<0.01 and ***; P<0.001). IQ; Isoquercetin and MM; Memantine.

The probe trial data analysis was performed in experimental rats. Colchicine-treated rats exhibited a significantly decreased latency towards the target quadrant as compared with the spend time by the control group. Moreover, such down-regulation in the time spent in the quadrant was enhanced upon the treatment with isoquercetinin a dose-dependent manner. A similar results were obtained in the memantine-treated group (Fig.5).

### Effect of isoquercetin on memory and learning via passive avoidance paradigm

**Fig.6:**
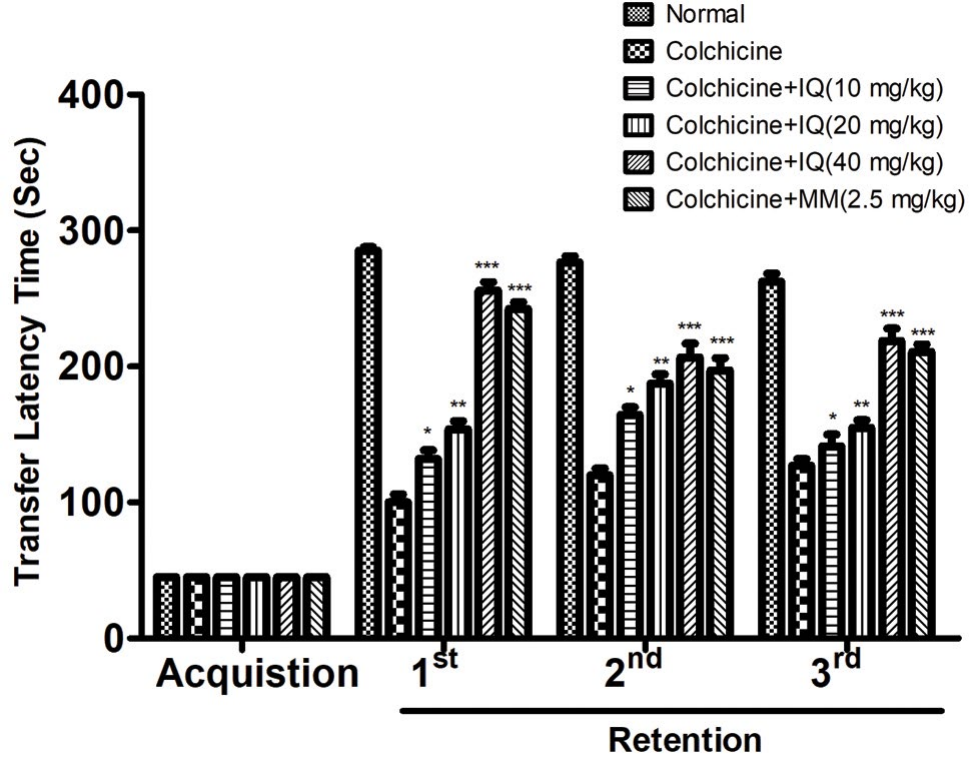
showed the effect of isoquercetin and colchicine on passive avoidance response; data shows mean ± SEM of 6 rats in each group. Significant difference represent as compared to colchicine control group rats (*; P<0.05, **; P<0.01, and ***; P<0.001). IQ; Isoquercetin and MM; Memantine

The passive avoidance paradigm was used for the estimated effect of isoquercetin on the memory and learning capacity of the rats. Colchicine induced rats exhibited brain damage as compared to the control group rats. Figure 6 showed that the reduced transfer latency time as compared to the acquisition trial transfer latency time from the control group to the colchicine-treated group (3^rd^ retention trial). The isoquercetin-treated group exhibited the increased transfer latency time as compared with the acquisition trial. On the other hand, the standard drug-treated group showed the increased transfer latency time as compared to the acquisition trial. Moreover, instantaneous isoquercetin received rats exhibited a significant enhance in TLT in retention trials in comparison with the acquisition trial transfer latency time.

### Effect of isoquercetin on brain-derived neurotrophic factor

Figure S1 (See Supplementary Online Information at www.celljournal.org) showed the effect of isoquercetin on the level of brain-derived neurotrophic factor (BDNF) in colchicine induced group rats. Colchicine induced group rats exhibited a reduced level of BDNF in comparison to normal control. Colchicine-induced group exhibited a significantly (P<0.001) increased level of BDNF in a dose-dependent manner. On the contrary, Memantine significantly (P<0.001) enhanced the level of BDNF, but the level was slightly lower as compared to the isoquercetin (40 mg/kg) treated drugs.

### Effect of isoquercetin on Aβ peptide activity

During the colchicine induced AD, the level of Aβ peptide activity considerably boosted and reached almost 2-3 times as compared to the normal control. A similar result was obtained in the colchicine induced treated group rats. The level of Aβ peptide activity reached almost 4 times more as compared to normal control. The dose-dependent treatment of isoquercetin treatment significantly (P<0.001) down-regulated the level of Aβ peptide activity as compared to the colchicine induced group rats. Standard drug (memantine) treated group rats showed the reduced level of Aβ peptide activity as compared to the colchicine induced group rats (Fig.S2) (See Supplementary Online Information at www. celljournal.org). 

### Effect of isoquercetin on acetylcholinesterase activity

Figure S3 (See Supplementary Online Information at www. celljournal.org) demonstrated the effect of isoquercetin on the level of acetylcholinesterase (AchE) on the colchicine induced group rats. Colchicine-induced group showed the reduced activity of AchE, while the dose-dependently treatment of isoquercetin and standard drug (memantine) significantly (P<0.001) increased the activity of AchE. 

### Effect of isoquercetin on P. carbonyl activity

Figure S4 (See Supplementary Online Information at www.celljournal.org) illustrated the effect of isoquercetin on the level of P. carbonyl activity. Colchicine induced group rats showed the increased activity of P. carbonyl activity and dose-dependently treatment of isoquercetin and standard drug (memantine) significantly (P<0.001) decreased the activity of P. carbonyl activity.

### Effect of isoquercetin on pro-inflammatory cytokines

Figure S5 (See Supplementary Online Information at www.celljournal.org) showed the effect of isoquercetin on the level of pro-inflammatory cytokines in the colchicine induced AD rats. Colchicine induced group rats showed an increased level of pro-inflammatory cytokines, such as TNF-α, IL-1β, and IL-6 and dose-dependent treatment of isoquercetin significantly (P<0.001) reduced the level of pro-inflammatory cytokines.

### Effect of isoquercetin on NF-κB activity

The level of NF-κB considerably boosted during AD. A similar result was obtained in the colchicine induced AD control group rats. The dose-dependent treatment of isoquercetin significantly (P<0.001) reduced the level of NF-κB as compared to the colchicine induced AD control group rats (Fig.S6) (See Supplementary Online Information at www.celljournal.org). 

## Discussion

In the year of 1976, PC12 cell lines have been considered as the significant model system for neurochemical and neurobiological investigations, as its adaptability, comfort culture, and huge information of their differentiation and proliferation (19). In the current investigation, we used the PC12 cells to investigate the neuroprotective effect of isoquercetin. In an *in vitro* model, PC12 cells were treated with the LPS, which is commonly present in the Gram-Negative bacteria membrane (22). By scrutinizing the apoptosis, cell viability, antioxidant and pro-inflammatory cytokines, we established the cell model of LPS was successfully constructed (23). It is well defined that oxidative stress plays an important mechanism underlying PC12 induced neurotoxicity during AD (24). Consequently, targeting oxidative stress is the best approach to treat A0. In the current experimental study, we established the LPS induced oxidative model in the PC12 cells. LPS significantly increased the level of MDA (LPO parameter) and intracellular formation of ROS and reduced the levels of SOD, CAT in the PC12 cells as compared with the non-stimulated cells, approaching the pathophysiological condition of oxidative stress during the central nervous system (CNS) disorders. Isoquercetin treatment significantly reduced the intracellular ROS generation, MDA level, and nitrite release and increased the activity of SOD, GSH, and CAT, suggesting the antioxidant role and maintain the intracellular oxidative stress and consequent neuroprotection. On the basis of the results, isoquercetin could be useful for the treatment of neuro-inflammatory diseases like AD, multiple sclerosis, PD etc. 

Neurodegenerative disease such as AD is considered as the age-related disease (25). It is related to the early phase of cognitive dysfunction linked with behavioural and social deterioration. The feature of AD is extracellular senile plaques, loss of neurons, intracellular neurofibrillary in the region of brain, such as hippocampus region (6). Previous research suggests that the hippocampus region of the brain is the primary target, which involved in AD pathophysiology (6, 9). It is well known that the accumulation of β amyloid and the generation of amyloid plaques are the hallmarks of AD (6, 26). In the current protocol, we made an attempt to scrutinize the neuroprotective effect of isoquercetin against the *in vitro* and* in vivo* model of AD and explore the possible mechanism of action. 

For the learning and memory impairment, colchicine induced AD model is commonly used (27). Colchicine induced the dysfunction in various parts of the brain, especially when inducing the severity into the hippocampus region of the brain. It is well known that the level of AChE was reduced during the impaired learning and memory capacity of rodents (28). A similar result was found in the colchicine-induced group; a marked reduced AChE content was observed, while the dose-dependent treatment of isoquercetin significantly increased the AChE content and suggests the memory and learning improvement. Previous research suggests that the marked increase in the activity of AChE and decrease the activity of choline acetyltransferase showed a reduced level of Ach (29). Various experimental study suggests that the decreased level of AChE activity, indicating the impairment of cognitive function and improved the activity of AChE suggests the increase cognitive function (27). Other parameters such as Aβ start the deposition during the AD in the cerebral area of the brain, and it induces the memory dysfunction with marked cholinergic function (30)bisdemethoxycurcumin and demethoxycurcumin. During AD, the accumulation of Aβ considerably increased and induced memory dysfunction. In the current experimental study, the content of Aβ was considerably boosted and dose-dependent treatment of isoquercetin significantly reduced the content of Aβ. A similar momentum was observed in the memantine induced group rats.

Previous research suggests that the colchicine administration exhibit the dose- and time-dependent behavioural, neurochemical, anatomical changes, and the changes reached upwithin 2-3 weeks (27). Colchicine (tubulin inhibitor), avoids microtubule assembly inducing synaptic loss and neurofibrillary degeneration that take part in the diminishing of intracellular trafficking of neurotrophic factors, oxidative stress, inflammation, and axonal excitotoxicity (31). Colchicine also disrupts the cytoskeleton that has to be linked with the neurodegeneration in AD and also executing a deadly effect on the activity and persistence of neurons (19). Meanwhile, colchicine administration does not produce a significant alteration in the gross locomotor and behavioural activities in rats. The current experimental study showed that the open field did not show higher scores for the locomotor and behavioural activities in each rat. 

Previous research suggests that the BDNF is the main target in the pathophysiology of various neurodegenerative diseases (19). BDNF is considering as the prognostic and diagnostic biomarker of AD. During the AD, the level of BDNF considerably reduced in the rodent model when treated with colchicine, and it might take part in the reduction of the hippocampus region of the brain linked with the age-related memory decline in the late adulthood (32). Studies suggest that the BDNF gene is related to the late onset of AD. To identify the pathophysiology of the neurodegenerative disease, the rodent model is the crucial tool, and most of researchers used the animal models for elucidation of the neurological disease pathophysiology (33, 34). One of these experimental models, central injection of colchicine, was injected into the lateral ventricles, which is measured as appropriate cases of sporadic dementia of Alzheimer’s in humans. During the AD, the level of BDNF was considerably reduced in the hippocampal tissue as compared to the untreated rats and dose-dependently treatment of isoquercetin significantly increased the level of BDNF suggesting the neuroprotective effect via neurotrophin induction. Therefore, the ultimate goal of the current study was elucidating the possible neuroprotective effect of isoquercetin in the animal model of AD disease and explore the possible underlying mechanism. 

During the inducing the colchicine intracerebroventricular (icv), its start the reduction of BDNF level into the hippocampal tissue, decreased the level of Aβ peptide level into the hippocampal tissue as well as down-regulated the antioxidant enzymes, these parameter suggesting that the colchicine injection deteriorates the memory and learning ability (34, 35) and its also induces the oxidative stress, boost the inflammatory reaction as well as induces the injury in the central neuronal. Restoration of these enzymes and parameters via isoquercetin suggesting the neuroprotective effect via enhancing the cognitive function. Definitely, this current experimental study showed the significant memory dysfunction in the Morris water maze test, as confirmed by considerably enhanced the initial acquisition latency, as well as 1^st^ and 2^nd ^retention latencies. 

Previous research suggests that the oxidative stress play a significant role in the expansion of AD (34, 35). Various studies suggest that oxidative stress related to ROS generation and take widely precipitation during the neurological and psychiatric disorder. During the induction of oxidative stress, frequently observed the imbalance between the pro-oxidant and endogenous antioxidant and its can be estimated via measured the redox state in the plasma. Endogenous antioxidant parameters such as SOD, CAT and GSH play a significant role to scavenge the free radicals (35, 36). During the colchicine induced AD, the level of free radical increase due to the induction of oxidative stress and reduced the level of endogenous antioxidant mechanism via increased the peroxidation (36, 37). CAT and SOD are considered as the first line antioxidant and both endogenous antioxidants scavenge the free radical especially the hydroxyl radicals (37, 38). Another antioxidant malonaldehyde (MDA) is the marker of lipid peroxidation and its take part in the oxidative stress and its also consider as the end product of the polyunsaturated fatty acid (PUFA) lipid peroxidation. The level of MDA significantly increased during the oxidative stress and its consider as the significant marker to estimate the oxidative stress throughout the body (37). During the experimental protocol, colchicine induced AD rats showed the increased level of MDA and isoquercetin significantly reduced the level of MDA at dose dependent manner. In the current protocol, the up-regulation of MDA and protein carbonyls level and down-regulation of CAT, GSH and SOD level were observed and dose dependent treatment of isoquercetin significantly reduced the level of MDA, protein carbonyls and increased the level of CAT, GSH and SOD, which suggest the reduction of oxidative stress in the brain. Result suggests the neuroprotective effect of isoquercetin via antioxidant nature.

Another factor of AD pathogenesis, inflammatory, and oxidative stress may take part in the expansion of the disease. The presence of the NF-κB was thefirsttime recognized in the nuclear B cell. NF-κB is the significant transcription factor, which contributes to the activation of genes that are involved in the generation of pro-inflammatory cytokines and induces neuroinflammation in AD. Several references suggest that the NF-κB, having the ability to merge the sequence-specific enhances of the immunoglobulins K light chain gene. During the generation of AD, the NF-κB level was increased in the senile plaques. Various research suggests that the activation of NF-κB and NF-κBp65 either directly or indirectly associated with the severity of AD and targeting the NF-κB is the best approach to treat AD. Isoquercetin already reported to reduce the NF-κB activation in the tumour cells and also induce the anti-inflammatory effect via suppressing the NF-κB activation in the lymphocytes (32). NF-κB activates pro-inflammatory cytokines such as IL-1β, IL-6, and TNF-α, which take part in the expansion or progression of inflammatory disease. It is well documented that neuro-inflammation play a crucial role in the expansion of neurodegenerative diseases, such as AD. Neuro-inflammation is involved in the microglial cells activation and also takes part in the participation of astrocytes and neurons. Recent research suggests that the pro-inflammatory cytokines such as IL-1β, IL-6, and TNF-α activate neuroinflammation and start the cognitive function destruction (27). Previous research suggests that the continuous generation of pro-inflammatory cytokines leads to the impairment of cognitive function in the brain. TNF-α, secrete from the plaques during the AD disease and also boost the secretion of IL-1β from the central nervous system. The up-regulation of IL-1β level in the hippocampus region showed the interfering of long-term potentiating that induce cognitive impairment (21, 26, 27). It also induced the suppression of the long-term potentiating, inducing the synaptic plasticity dysfunction in the hippocampus region of the brain. IL-1β also reduces the level of BDNF, which is indirectly reduced by LTP and also causes cognitive dysfunction. It is well documented that enhanced levels of pro-inflammatory cytokines, such as TNF-α and IL-1β in the hippocampus region of the brain are responsible for the dysfunction of postoperative cognitive (8). In the current protocol, isoquercetin significantly reduced the level of pro-inflammatory cytokines and suggestion the anti-inflammatory effect.

Previous research suggests that oxidative stress plays a significant role in the expansion of AD. Various studies suggest that oxidative stress and ROS generation are evident during the neurological and psychiatric disorders. Continuous generation of oxidative stress can induce apoptosis and cell death. During the induction of oxidative stress, frequently observed the imbalance between the pro-oxidant and endogenous antioxidants, and it could be estimated via measuring the redox state in the plasma. CAT and SOD are considered the first-line antioxidants, and the both endogenous antioxidants scavenge the free radicals, especially hydroxyl radicals (37, 38). The endogenous antioxidants, such as SOD, CAT, and GSH play a significant role in scavenging the free radicals (39, 40). During colchicine-induced AD, the level of the free radicals are increased due to the induction of oxidative stress and reduced the level of the endogenous antioxidant mechanism via increasing the peroxidation (38, 39). Another antioxidant MDA is the marker of lipid peroxidation, and it participates in oxidative stress, and it is also considered the end-product of the polyunsaturated fatty acid (PUFA) lipid peroxidation. The level of MDA significantly increased during the oxidative stress and it is considered a significant marker to estimate oxidative stress throughout the body (27). During the experimental protocol, colchicine-induced AD showed an increased level of MDA, while isoquercetin significantly reduced the level of MDA in a dose-dependent manner. In the current protocol, the up-regulation of MDA and protein carbonyls level and down-regulation of CAT, GSH, and SOD level were observed. The dose-dependent treatment of isoquercetin significantly reduced the level of MDA, protein carbonyls, and it increased the level of CAT, GSH, and SOD, suggesting the reduction of oxidative stress in the brain. The results suggest the neuroprotective effect of isoquercetin via antioxidant nature. 

## Conclusion

Isoquercetin did not demonstrate the impact of cell viability on the PC12 cells. PC12 cells treated with LPS had increased nitrile, and ROS levels, and the dose-dependent isoquercetin treatment reduced the level of oxidative stress. The dose-dependent treatment of isoquercetin significantly altered the antioxidant, and pro-inflammatory parameter. Colchicine decreased the latency period and significantly increased the latency time and dose-dependent treatment of isoquercetin in the *in vivo* experimental study. Colchicine-induced group rats decreased levels of BDNF and AchE, while significantly increased levels of Aβ-peptide, P. carbonyl and dose-dependent isoquercetin treatment increased levels of BDNF, AchE and decreased levels of Aβ-peptide, P. carbonyl. Colchicine-induced group rats increased the level of pro-inflammatory cytokines and inflammatory mediators and significantly reduced the level of pro-inflammatory cytokines and inflammatory mediators through dose-dependent isoquercetin treatment. The result showed that isoquercetin significantly altered cognitive function and prevented neurochemical and neurobehavioral alteration against colchicine by inducing rats of AD through the antioxidant and anti-inflammatory mechanism.

## Supplementary PDF


