# Supplementary material for: Neuroprotective Effects of Isoquercetin: An In Vitro and In Vivo Study
Source: Cell J. 2021 Jul 17;23(3):355–65. doi: 10.22074/cellj.2021.7116 (PMC8286454; doi:10.22074/cellj.2021.7116)
Supplement: Supplementary file 1 [file Cell-J-23-355-s01.pdf]

CELL JOURNAL (Yakhteh)

Supplementary Information for

Neuroprotective Effects of Isoquercetin: An *In Vitro* and *In Vivo* Study

Qingxiao Yang, M.M.[1], Zhichen Kang, M.D.[2], Jingze Zhang, M.M.[1], Fuling Qu, M.M.[2], Bin Song, M.M.[1*]

1. Neurosurgery Department, Second Hospital of Jilin University, Changchun City, Jilin Province, 130000, China
2. Rehabilitation Department, Second Hospital of Jilin University, Changchun City, Jilin Province, 130000, China

***Corresponding Address: Neurosurgery Department, Second Hospital of Jilin University, Changchun City, Jilin Province, 130000, China**
Email: 15526859189m0@sina.cn

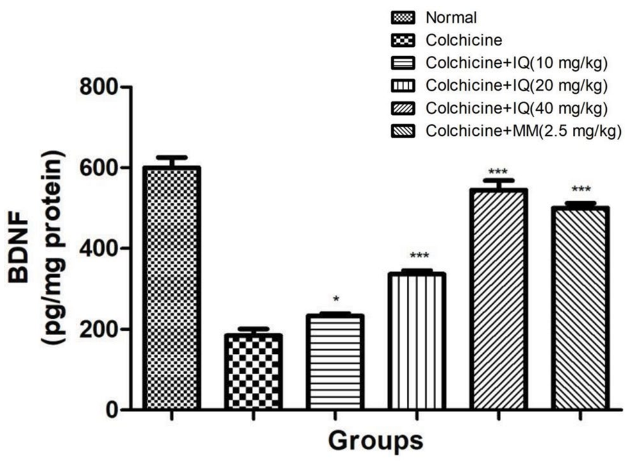

Fig.S1: Showed the effect of isoquercetin and colchicine on the level of BDNF. Method as described in the material and method. Data shows mean ± SEM of 6 rats in each group. Significant difference represent as compared to colchicine control group rats. *; P<0.05, ***; P<0.001, BDNF; Brain-derived neurotrophic factor, IQ; Isoquercetin, and MM; Memantine.

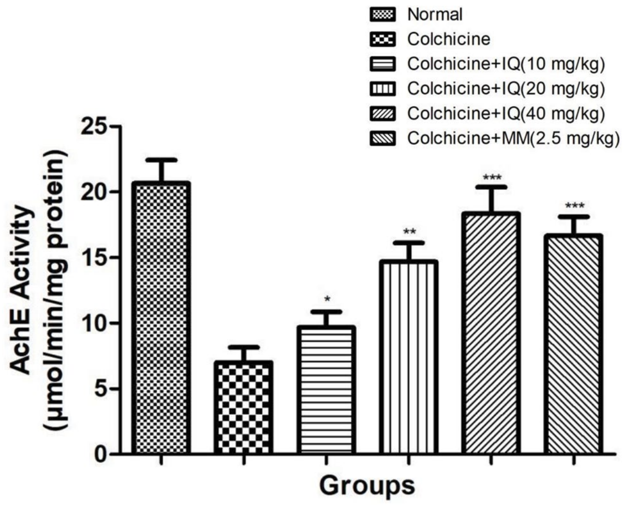

Fig.S3: Showed the effect of isoquercetin and colchicine on the level of AchE activity. Method as described in the material and method. Data shows mean ± SEM of 6 rats in each group. Significant difference represent as compared to colchicine control group rats. *; P<0.05, **; P<0.01, ***; P<0.001, IQ; Isoquercetin, and MM; Memantine.

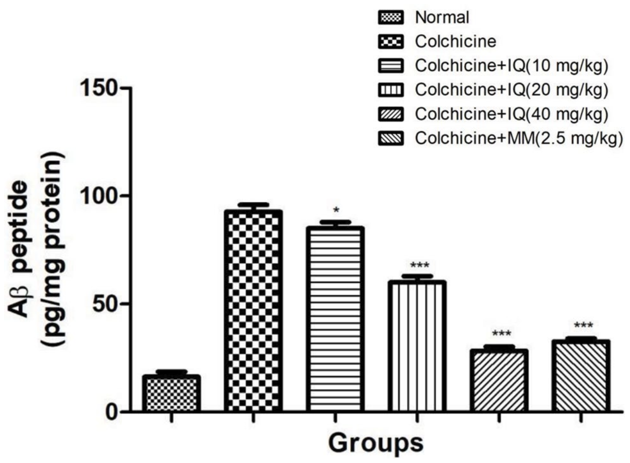

Fig.S2: Showed the effect of isoquercetin and colchicine on the level of Aβ peptide. Method as described in the material and method. Data shows mean ± SEM of 6 rats in each group. Significant difference represent as compared to colchicine control group rats. *; P<0.05, ***; P<0.001, IQ; Isoquercetin, and MM; Memantine.

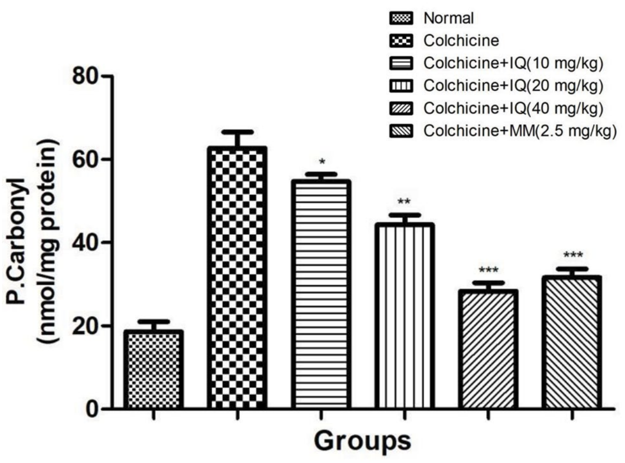

Fig.S4: Showed the effect of isoquercetin and colchicine on the level of protein carbonyl. Method as described in the material and method. Data shows mean ± SEM of 6 rats in each group. Significant difference represent as compared to colchicine control group rats. *; P<0.05, **; P<0.01, ***; P<0.001, IQ; Isoquercetin, and MM; Memantine.

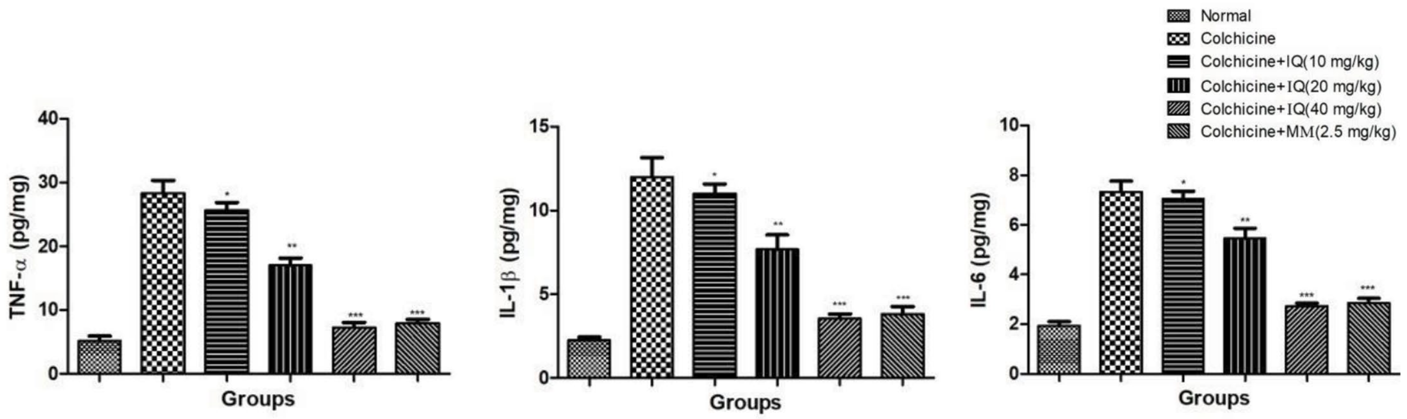

Fig.S5: Showed the effect of isoquercetin and colchicine on the level of pro-inflammatory cytokines. Method as described in the material and method. Data shows mean ± SEM of 6 rats in each group. Significant difference represent as compared to colchicine control group rats. *; P<0.05, **; P<0.01, ***; P<0.001, IL-1β; Interleukin-1β, TNF-α; Tumor necrosis factor-α, IL-6; Interleukin-6, IQ; Isoquercetin, and MM; Memantine.

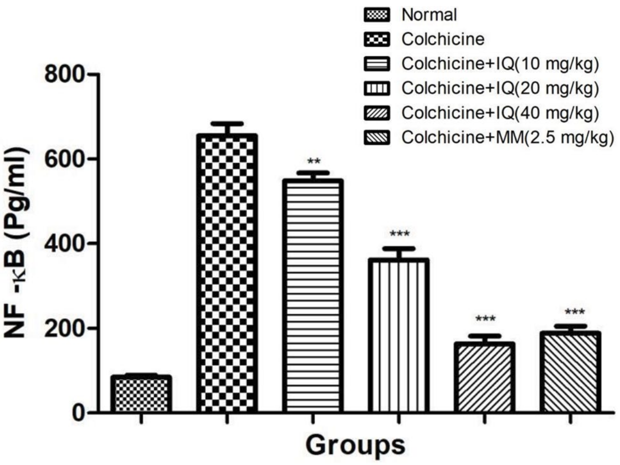

Fig.S6: Showed the effect of isoquercetin and colchicine on the level of NF-κB. Method as described in the material and method. Data shows mean ± SEM of 6 rats in each group. Significant difference represent as compared to colchicine control group rats. **; P<0.01, ***; P<0.001, TNF-α; Tumor necrosis factor-α, IQ; Isoquercetin, and MM; Memantine.
